# Phenotypic and Genomic Divergence in Biofilm Formation in STEC and Non-STEC *Escherichia coli*: The Impact of Genomic Plasticity on the Virulence and Persistence of Bovine Isolates

**DOI:** 10.3390/pathogens15080807

**Published:** 2026-07-31

**Authors:** Vinicius Silva Castro, Emmanuel W. Bumunang, Xianqin Yang, Yuri Duarte Porto, Eduardo Eustáquio de Souza Figueiredo, Kim Stanford

**Affiliations:** 1 Department of Biological Sciences, University of Lethbridge, Lethbridge, AB T1K 3M4, Canada; vinicius.castro1@ufmt.br (V.S.C.); emmanuel.bumunang@uleth.ca (E.W.B.); 2 Faculty of Nutrition, Federal University of Mato Grosso (UFMT), Cuiabá 78060-900, MT, Brazil; ydporto@ufrrj.br (Y.D.P.); eduardo.figueiredo@ufmt.br (E.E.d.S.F.); 3 Agriculture and Agri-Food Canada Research and Development Centre, Lacombe, AB T4L 1V8, Canada; xianqin.yang@agr.gc.ca; 4 Faculty of Agronomy and Zootechnics, Federal University of Mato Grosso (UFMT), Cuiabá 78060-900, MT, Brazil

**Keywords:** Shiga toxin-producing *Escherichia coli*, biofilm, whole-genome sequencing, mobile genetic elements, antimicrobial resistance

## Abstract

Shiga toxin-producing *Escherichia coli* (STEC) is a zoonotic pathogen of global relevance whose persistence in food processing environments is frequently mediated by biofilm formation. The acquisition of *stx* prophages and associated regulatory mutations can impose adaptive restrictions on this phenotype. This study analyzed 216 whole-genome sequences (WGS) to investigate the genomic determinants of biofilm formation in a diverse collection of *E. coli* isolates (STEC and non-STEC) isolated from cattle feedlots. Genomes were characterized for serogroup, MLST, *stx* subtyping, integrity of the *mlrA* and *rpoS* regulators, single nucleotide polymorphisms (SNPs) in the *csg*/*bcs* operons, antimicrobial resistance (AMR) genes, and plasmid replicons. The biofilm phenotype was quantitatively evaluated using a crystal violet assay at 15 °C for 96 h. Results demonstrated higher biofilm forming ability among non-STEC isolates compared to STEC strains, with strains simultaneously carrying both *stx1* and *stx2* exhibiting the lowest prevalence of biofilm formation (3.3%). Although the *rpoS* mutation did not show a significant overall association (*p* = 0.354) with biofilm formation, it was universally present across all O157:H7 isolates, and likely enhanced *mlrA* disruption toward *csgD* repression. In addition, *stx*-positive isolates accumulated significantly more SNPs in the curli (*csg*) and cellulose (*bcs*) structural genes. Exploratory gene-level association tests and multiple correspondence analysis further indicated that *stx* status was associated with broader differences in non-*stx* accessory gene composition. In addition, there was no statistical association between AMR classes and biofilm-forming capacity. Furthermore, STEC strains demonstrated a lower frequency of resistance to aminoglycosides, phenicols, and tetracyclines. Finally, the plasmid replicons IncX1 and IncFII(pSE11) were present and associated with biofilm-positive isolates. In conclusion, biofilm suppression in STEC suggests a multifactorial and evolutionary phage-induced regulatory trade-off, whereas biofilm persistence in non-STEC strains is independently driven by mobile genetic elements and potential accessory determinants.

## 1. Introduction

Shiga toxin-producing *Escherichia coli* (STEC) is a zoonotic pathogen of global relevance, triggering clinical symptoms varying from watery diarrhea to more severe conditions, such as hemorrhagic colitis and hemolytic uremic syndrome (HUS) [1]. Cattle are the primary reservoir of STEC, and transmission to humans occurs predominantly through the consumption of undercooked beef, unpasteurized dairy products, and contaminated vegetables and water, besides potential cross-contamination [2]. Hundreds of STEC serogroups have been identified, with O157:H7 historically the most closely associated with outbreaks and severe cases in humans; however, other non-O157 serogroups (principally O26, O103, O111, O121, O145, and O104) account for an increasing number of reported infections across different areas worldwide [3].

Virulence and strain classification of STEC is attributed to the presence of Shiga toxins (Stx), encoded by the *stx1* and *stx2* genes, which are carried by prophages integrated into the bacterial chromosome [4]. Likewise, there is a wide diversity of *stx* subtypes, including *stx1a*, *stx2a*, and *stx2c*, among others, with subtypes associated with differences in virulence potential. Among these subtypes, *stx2a* is consistently linked to the most severe clinical consequences, such as HUS, and is widely present in animal isolates [5]. Although Shiga toxin is the primary pathogenic feature, other systems, such as the locus of enterocyte effacement (LEE), which encodes a type III secretion system (T3SS), and virulence plasmids such as pO157, contribute to the adherence, colonization, and pathogenicity of STEC isolates [6]. Also, the high genomic plasticity of *E. coli*, demonstrated by the dynamic acquisition and loss of *stx* phages among strains of different serogroups results in both toxigenic and non-toxigenic isolates sharing classical STEC serogroups, posing a major challenge for both epidemiological surveillance and food safety risk assessment [7].

Within this context, biofilm formation is a critical determinant of bacterial persistence in food processing environments, slaughterhouse surfaces, and industrial equipment [8]. Biofilms provide cells with protection against sanitizing agents, desiccation, oxidative stress, and antimicrobial treatments, thereby increasing the risk of cross-contamination throughout the production chain [9]. In *E. coli*, biofilm production is regulated by a complex transcriptional network primarily driven by *csgD*, which coordinates the synthesis of curli fibers and cellulose, the primary components of the extracellular matrix, encoded by the *csgBAC* and *bcsABCZEFG* operons, respectively [10,11]. The activation of the *csgD* gene is further dependent on the transcriptional regulator *mlrA*, the stationary-phase sigma factor rpoS, and the second messenger c-di-GMP, among other environmental signals [12,13].

A key aspect of STEC pathogenicity lies in the relationship between *stx* prophage acquisition and biofilm-forming capacity. Previous studies have demonstrated that the integration of the *stx* prophage into the *mlrA* gene, as a preferred integration site in serogroup O157:H7, functionally disrupts this regulator, thereby repressing the *csgD*-dependent cascade and resulting in a low-biofilm-forming phenotype [14]. Furthermore, mutations in the *rpoS* gene, which are universally present in O157:H7, reinforce this regulatory block on biofilm formation [15]. This combination of mechanisms suggests a potential evolutionary trade-off: while the acquisition of toxigenic phages may confer enhanced acute virulence to STEC strains, it may also come at the cost of suppressing biofilm-mediated environmental persistence mechanisms. Conversely, spontaneous excision of the prophage from the *mlrA* locus reverses this phenotype, restoring curli and biofilm production capacity, which suggests that this trade-off is not absolute and can be dynamically modulated [16].

An important point is that non-STEC isolates constitute a highly heterogeneous group encompassing diverse pathotypes as well as commensal strains. If STEC strains lose the *stx*-encoding prophage through excision, they frequently maintain an intact *mlrA* gene and exhibit greater biofilm-forming capacity compared to their toxigenic counterparts [16]. Considering that non-STEC isolates can share the same clinically relevant serogroups (O157, O26, O121, O145, O103) with STEC isolates, they may represent potential persistent reservoirs on processing surfaces, the identification and control of these population may be underestimated by detection methods generally focused on screening solely for the presence of the *stx* gene.

In addition to chromosomal biofilm regulatory pathways, mobile genetic elements can confer an additional and partly independent biofilm-forming capacity by harboring accessory genes for adhesion, exopolysaccharide synthesis, or c-di-GMP regulators [17]. The association between specific plasmid replicons and biofilm phenotypes has been reported in other *E. coli* pathotypes and across *Enterobacteriaceae* broadly, particularly for IncF and IncX plasmids that harbor fimbrial operons and other plasmid-encoded determinants [18,19].

Whole-genome sequencing (WGS) represents the most comprehensive tool to address these questions in an integrated manner, enabling the simultaneous characterization of serogroups, MLST, biofilm structural and regulatory genes, plasmids, and AMR genes within a single analytical platform [20]. Furthermore, the analysis of accessory genome is essential for understanding how restricted-distribution accessory genes can contribute to adaptive phenotypes such as biofilm formation in bacterial populations with highly diverse genomic backgrounds [21].

The present study utilizes WGS to characterize a diverse collection of *E. coli* serogroups originating from cattle farms, including both STEC and non-STEC strains, to investigate the genomic determinants of biofilm formation within this context. This study aimed to: (i) evaluate the association between toxigenic status (*stx*) and biofilm-forming capacity, as well as its underlying molecular regulatory mechanism(s); (ii) characterize the impact of prophage integration into the *mlrA* locus and the mutational status of *rpoS* on the biofilm phenotype across a diverse range of serogroups and sequence types (STs); (iii) investigate SNP variations within curli and cellulose structural genes between *stx*-positive and *stx*-negative isolates as an indicator of differential selective pressure; (iv) identify biofilm-related genes and explore multivariate patterns of non-*stx* gene composition associated with *stx* status; and (v) determine the plasmid replicons and AMR genes associated with the biofilm phenotype.

## 2. Materials and Methods

### 2.1. Isolate and Sequencing Data

This study applied a quantitative, retrospective, and analytical approach, where the inclusion criterion for the 216 *E. coli* isolates analyzed was their origin from our previous studies of feedlot cattle from a variety of locations in southern Alberta (Canada) [22]. Among those included in the dataset, 186 isolates had their genomic sequences obtained from previous evaluations that examined different variables, such as the association between *E. coli* serogroups and resistance to quaternary ammonium compounds and organic acids [23], inconsistencies in the PCR-based detection of Shiga toxin-producing *Escherichia coli* [24], genomic characterization of *E. coli* O157:H7 strains from presumably super- and low-shedding cattle [25], as well as from experiments that evaluated the efficacy of quaternary ammonium compounds against *E. coli* strains with high and low tolerance to these agents [26]. The remaining 30 isolates from collection have their whole-genome sequences published for the first time in the present study and these additional isolates were most recent in origin (2024 and 2025) compared to the previously sequenced isolates which were originally collected between 2007 and 2015. All isolates were evaluated for biofilm phenotype as described in Section 2.2, regardless of whether they had been tested in previous studies.

The genomic data for all isolates used in this study are publicly available under NCBI BioProjects: PRJNA1166229, PRJNA1038721, PRJNA601484, PRJNA870153, and PRJNA846735. Genomic DNA was extracted from cultures incubated in 5 mL of Luria–Bertani (LB) broth using the DNeasy Blood & Tissue Kit (QIAGEN, Toronto, ON, Canada) according to the manufacturer’s instructions. Whole-genome sequencing was performed on an Illumina NovaSeq 6000 platform, generating 150 bp paired-end reads with an estimated coverage depth greater than 300× per genome. Raw read quality was assessed using FastQC v0.11.7, and adapter sequences. Low-quality bases were removed using Trimmomatic v0.39 with default parameters [27]. De novo assemblies were generated using SPAdes v3.14.0, with optimized k-mer values for each genome [28], and assembly quality was assessed using QUAST v5.0.2 [29]. All subsequent genomic analyses described below were performed on these curated assemblies.

### 2.2. Biofilm Production Assay

Biofilm formation was assessed using a crystal violet assay in 96-well microtiter plates with peg lids, following a protocol adapted from Ma et al., [30] and Stanford et al., [31]. Briefly, isolates were recovered from frozen glycerol stocks onto MacConkey agar (Daylynn Biologicals, Calgary, AB, Canada) and incubated overnight at 37 °C. Single colonies were picked and inoculated into Tryptic Soy Broth (TSB; MilliporeSigma, Oakvile, ON, Canada) and grown with shaking (200 rpm) to obtain standardized cultures. These cultures were then diluted 1000-fold in fresh TSB to achieve a cell density of approximately 10^7^ CFU/mL, and 200 µL of the diluted suspension was dispensed into individual wells of sterile, flat-bottom, 96-well polystyrene microtiter plates (Thermo Fisher Scientific, High River, AB, Canada). Peg lids were fitted to allow biofilm formation. Negative control wells containing only sterile TSB were included in each plate.

The plates were incubated under static conditions at 15 °C for 96 h to allow biofilm development on the polystyrene surface. Following incubation, planktonic cells and spent medium were carefully removed, and the wells were washed three times with sterile phosphate-buffered saline (PBS) to remove non-adherent bacteria. The remaining adhered biomass was fixed by air-drying and stained with 0.1% (*w*/*v*) crystal violet (Millipore Sigma) for 20 min. Excess dye was removed by washing twice with PBS (Thermo Fisher Scientific). Bound crystal violet was then solubilized with 80% ethanol, and absorbance was measured at 570 nm using a microplate reader (Tecan Austria GmbH Untersbergstr. 1A A-5082 Grodig, Austria).

For each isolate, biofilm production was quantified as the mean OD570 value of the technical replicates after subtracting the negative control (blank) from the same plate. Isolates were classified as either non-biofilm formers or biofilm formers. To determine positive biofilm formation, a cutoff threshold was established at greater than three times the value of the negative control.

### 2.3. E. coli Serogroup, MLST and STEC Determination

In silico serotyping of *E. coli* was performed on assembled genomic sequences using ABRicate (v1.4.0; https://github.com/tseemann/abricate; accessed on 13 January 2026) with the specialized EcOH database. This database provides reference sequences for the prediction of O and H antigen determinants [32]. Multilocus sequence typing (MLST) was performed using the mlst software (v2.11; Seemann T, https://github.com/tseemann/mlst; accessed on 29 Jaunary 2026), based on the Achtman seven-locus scheme for *E. coli* (*adk*, *fumC*, *gyrB*, *icd*, *mdh*, *purA*, and *recA*) accessed on via the PubMLST database [33]. Shiga toxin (*stx*) gene subtyping was performed using StxTyper (v1.0.45; https://github.com/ncbi/stxtyper; accessed on 10 February 2026), which classifies full-length *stx* operons via alignment against a curated reference database of StxA and StxB protein sequences from the NCBI Pathogen Detection Reference Gene Catalog, applying default thresholds of 60% sequence coverage and 80% sequence identity.

### 2.4. mlrA Status and SNP Determination for Curli and Cellulose Genes

The integrity of the *mlrA* gene, which encodes a transcriptional regulator required for *csgD* activation and, consequently, for the production of curli and the extracellular matrix [13], was evaluated using BLASTn (version 2.16.0) alignment of each assembled genome against the *mlrA* reference sequence obtained from the NCBI RefSeq database (genome NC_000913.3, protein accession NP_416631.1). In addition, mutations in the *rpoS* gene were also analyzed using the reference sequence (genome NC_000913.3, GeneID 947210). Sequences exhibiting premature stop codons, frameshift mutations, or nucleotide identity below 80% were classified as non-functional (*mlrA*-negative) or as carrying a mutation (*rpoS*-mutated).

To investigate whether prophage insertion within *mlrA* could account for gene absence or disruption, genomic context analysis was performed using the GFF3 annotation files generated by Bakta for each assembled genome. For each isolate in which *mlrA* was not detected, a sliding window of 15 annotated features surrounding the expected *mlrA* locus was examined for the presence of phage-associated gene annotations, including integrases, terminases, tail and capsid proteins, lambdoid elements, and transposases. Isolates presenting one or more such features in the immediate genomic neighborhood of the *mlrA* locus were classified as harboring a prophage proximal to *mlrA*, suggesting that phage integration may have contributed to gene disruption or displacement at this regulatory locus. 

To assess sequence diversity within the curli (*csgA*: GeneID: 949055; *csgB*: GeneID: 947391; *csgC*: GeneID: 945623; *csgD*: GeneID: 949119; *csgE*: GeneID: 945711; *csgF*: GeneID: 945622; and *csgG*: GeneID: 945619; and cellulose biosynthesis (*bcsA*: GeneID: 948053; *bcsB*: GeneID: 948045; *bcsC*: GeneID: 948047; *bcsE*: GeneID: 948050; *bcsF*: GeneID: 948059; *bcsG*: GeneID: 915719; *bcsQ*: GeneID: 948052; and *bcsZ*: GeneID: 948046) gene clusters, BLASTn searches were performed against all assembled genomes using reference sequences from genomes NC_002695 or NC_000913.3. The searches were conducted with minimum thresholds of 80% sequence identity and an E-value ≤ 1 × 10^−10^. For each gene-isolate pair, the top-scoring hit was retained, and the number of mismatches reported by BLAST was used as an indicator of the single nucleotide polymorphism (SNP) count relative to the reference sequence. The results were compiled into a presence/absence and SNP count matrix, and the associations between SNP counts and phenotypic traits, such as biofilm production and the presence of the Shiga toxin genes (*stx*), were independently evaluated for each gene.

### 2.5. Biofilm-Related Gene Determination

Biofilm-related genes were retrieved from the UniProtKB/Swiss-Prot database (https://www.uniprot.org; accessed on 17 March 2026) by searching for *Escherichia coli* proteins annotated with biofilm-associated terms. The search query used was “biofilm AND *Escherichia coli*”, filtering the results for “reviewed” status and selecting the *E. coli* K-12 (MG1655) reference proteome to obtain a high-confidence, non-redundant gene panel. This strategy yielded a curated list of 221 protein-coding genes with experimental or curated evidence of involvement in adhesion, extracellular matrix production, regulatory networks, or stress responses linked to *E. coli* biofilm formation. All gene sequences were downloaded in FASTA format and used in subsequent analyses.

Thus, a reference file containing the sequences of all 221 genes was used to screen all genomes in the dataset. Nucleotide similarity searches were performed using BLASTn (BLAST+), applying a minimum identity threshold of 80% and an E-value cutoff of 1 × 10^−10^ to detect putative homologs while accounting for allelic variation. For each gene-isolate pair, only the top-scoring hit (highest bit score) meeting the established thresholds was retained. Genes with at least one qualifying hit were recorded as present (value 1) in that genome, whereas those with no hits meeting the criteria were considered absent (value 0), thereby generating a binary presence/absence matrix for the 221 biofilm-related genes across all isolates.

This binary matrix was then compared against the phenotypic traits (biofilm production and *stx* status). To focus on patterns with potential biological relevance, the genes were filtered to retain only those exhibiting an absolute difference of at least 10% in presence frequency among predefined groups: (1) *stx*+, (2) *stx*+/biofilm+, (3) *stx*+/biofilm−, (4) *stx*−/biofilm−, (5) *stx*−/biofilm+, (6) all *E. coli*, and (7) *stx*−. The filtered gene set was visualized through hierarchical clustering and heatmaps using Seaborn’s clustermap in Python (version 0.13.2). Euclidean distance and Ward’s linkage method were applied to both genes and isolates, allowing the identification of genomic clusters that share similar biofilm-related gene repertoires or *stx* gene presence profiles.

### 2.6. In Silico Determination of Antimicrobial Resistance and Plasmid Content Determination

Antimicrobial resistance (AMR) genes were identified using ABRicate (v1.4.0) with the Comprehensive Antibiotic Resistance Database (CARD) as the reference database. For each assembled genome, ABRicate was run with default parameters, and only hits with a minimum nucleotide identity of 80% and a coverage threshold of 80% or greater were retained. The results were compiled into a binary presence/absence matrix of AMR genes per isolate. This matrix was then used to calculate the frequency of each AMR determinant among the phenotypic and genotypic groups of interest. Furthermore, the proportion of isolates harboring each AMR gene was compared between biofilm-producing and non-producing isolates, as well as between *stx*-positive and *stx*-negative isolates.

Plasmid replicon content was determined using PlasmidFinder on the assembled genomes. For each isolate, the PlasmidFinder results were exported in tabular format and subsequently analyzed to compile a list of incompatibility (Inc) groups and other replicon types detected within that genome. Replicons were considered present if they met the tool’s default thresholds for identity and coverage (a minimum of 80% for both identity and coverage), and absent otherwise. These results were then integrated into a replicon presence/absence matrix. Based on this matrix, plasmid replicon frequencies were calculated for biofilm-positive and biofilm-negative isolates.

### 2.7. Exploratory Association Tests and Multiple Correspondence Analysis

Statistical analyses were conducted to explore the distribution of the evaluated genes in relation to the presence of *stx* genes, biofilm production, and isolate serogroups. The complete dataset was used as the initial matrix to construct the analyses and was made available as Supplementary Dataset S1. Gene variables were treated as binary variables based on the absence or presence of each gene in the evaluated isolates.

Therefore, an initial assessment of the dataset was conducted, including the identification of metadata variables, binary gene variables, and the frequency of presence for each gene. This frequency was calculated by dividing the number of positive isolates for a given gene by the total number of evaluated isolates. For the Multiple Correspondence Analysis (MCA), only genes with a frequency of presence between 25% and 75% were retained, as this range was defined as an operational criterion to ensure variables possessed sufficient variability to contribute to the discrimination of gene profiles in the factorial plane. Genes that were either too rare or nearly constant were excluded solely from the active matrix of the multivariate analysis, while remaining described in the exploratory tables and the complete dataset. The gene presence frequencies, their classification based on the selection criterion, and the list of retained genes are presented in Supplementary Table S1.

In the Multiple Correspondence Analysis (MCA), *stx* presence was used as the primary supplementary grouping variable, classified as Stx_0 for *stx*-negative sequence and Stx_1 for *stx*-positive sequences. Genes starting with *stx* were not included as active MCA variables to avoid circular interpretation, since *stx* presence was projected as a supplementary grouping variable. Each active gene was transformed into a categorical variable with two modalities: absence (gene_0) and presence (gene_1). The final analytical matrix, containing the selected non-*stx* genes as active variables and the supplementary variables used for interpretation, was made available as Supplementary Dataset S2. In addition to the *stx*_presence variable, biofilm production and serogroup were also evaluated as supplementary exploratory variables to aid in the interpretation of the observed multivariate structure, without participating in the construction of the MCA dimensions. Associations between variables were evaluated using Pearson’s chi-square test or Fisher’s exact test (*stx*_presence × grouped serogroup and biofilm production × grouped serogroup), depending on the distribution of expected frequencies. Effect size was estimated using Cramér’s V. For 2 × 2 comparisons, prevalence ratio and odds ratio were estimated with their respective 95% confidence intervals, where applicable. For gene-level tests, *p*-values were adjusted using the Benjamini–Hochberg method. Full results are presented in Supplementary Table S3.

Additionally for the MCA, the first two dimensions were retained for graphical interpretation, based on the proportion of explained inertia and the distribution of modalities on the factorial plane. To assess the quality of representation and the structure of the data, the coordinates, contributions, and squared cosine values of the active modalities were computed, along with the projections of individual samples and supplementary categories. The complete results of the analysis are presented in Supplementary Table S4. For the final graphical representation, the modalities displayed in the biplot were objectively selected based on the factorial poles of the first two dimensions. For each pole, the modalities were ranked by a score calculated as the product of the absolute coordinate value in the respective dimension and the modality’s contribution to that same dimension. The seven modalities with the highest score in each pole were included in the final figure to preserve readability and represent the most relevant modalities of the multivariate structure. The selected modalities and their respective scores are presented in Supplementary Table S5. In the final biplot, isolates were represented according to their coordinates in the first two dimensions and grouped according to *stx* presence. Interpretation of the analysis was based on the proximity, opposition, and clustering of categories on the factorial plane, without inferring causality.

Analyses were performed using R software, version 4.4.2 [34]. The following R packages were utilized: readxl [35] for importing spreadsheet files; writexl [36] for exporting analytical and supplementary tables; dplyr [37], tidyr [38], tibble [39], and stringr [40] for data organization, filtering, and transformation; FactoMineR [41] for performing Multiple Correspondence Analysis (MCA); ggplot2 [42] for generating figures; and ggrepel [43] for label adjustment in plots. Functions from the base stats package were employed for chi-square tests, Fisher’s exact test, and *p*-value adjustment via the Benjamini–Hochberg method. Estimates for prevalence ratios, odds ratios, confidence intervals, and Cramér’s V were calculated within the analytical workflow based on the generated contingency tables.

### 2.8. Statistical Determinations

Fisher’s exact test was used to assess the associations between binary categorical variables, specifically the presence or absence of functional *rpoS* and *mlrA* genes, AMR genes, and plasmid replicons, and phenotypic groups (biofilm-positive vs. biofilm-negative; *stx*-positive vs. *stx*-negative).

A two-tailed Mann–Whitney U test was applied to compare the SNP counts per gene between phenotypic groups, as SNP counts represent discrete, non-negative integers derived from BLAST mismatch data and are not normally distributed. For all analyses, statistical significance was defined as *p* < 0.05.

## 3. Results and Discussion

### 3.1. Serogroups, Shiga Toxin-Presence, MLST and Biofilm Formation

**Figure 1:**
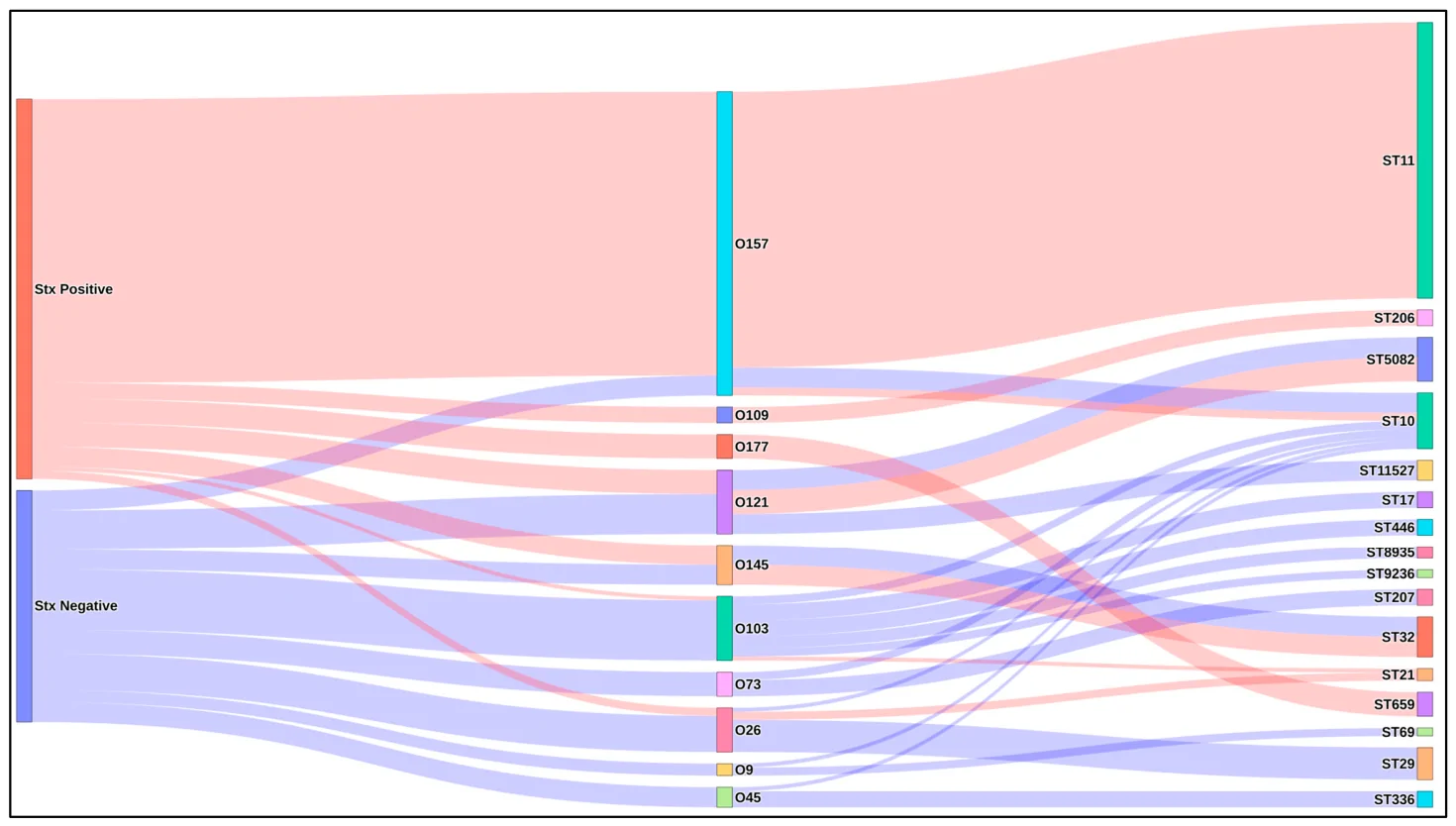
Distribution of Shiga toxin (*stx*) profiles, O serogroups, and sequence types (STs) in *Escherichia coli* isolates. **Legend:** Sankey diagram illustrating the phylogenetic and virulence relationships of the isolated collection. The flow maps the association among the presence or absence of Shiga toxin genes (*stx*-positive in red; *stx*-negative in blue; left column), the diversity of identified serogroups (middle column), and the respective sequence types (STs) determined by MLST (right column). The width of the horizontal bands is directly proportional to the number of isolates within each genomic cluster.

Serogroup analysis revealed high diversity, particularly among non-*stx* isolates (Figure 1). Although non-*stx* isolates may carry diverse pathogenicity mechanisms, the isolates evaluated in this study were originally recovered using STEC-specific isolation methodologies and were all from cattle. Consequently, the non-STEC strains in our dataset are likely to represent STEC that have either lost the *stx* prophage or never had *stx*, rather than other pathotypes. Among *stx*-positive isolates, a high prevalence of serogroup O157 linked to ST11 was observed, which is one of the leading serogroups associated with human disease cases and food contamination worldwide [44]. Another notable finding is the sharing of serogroups between *stx*-positive and *stx*-negative isolates, a phenomenon that highlights the highly dynamic acquisition and loss of bacteriophages and the genetic plasticity exhibited by *E. coli*. This is further evidenced by the presence of non-STEC strains sharing classic STEC serogroups, such as O157, O121, O145, O103, and O26 (Figure 1). Regarding the high diversity of STs associated primarily with non-*stx* strains, this finding indicates substantial genetic diversity among the isolates, representing a broad genetic reservoir from which varied genes with potential roles in biofilm formation may emerge.

Phenotypic biofilm analysis demonstrated a higher positivity rate among non-*stx* isolates compared to *stx*-carrying strains. Specifically, among the 108 *stx*-negative isolates, 17 (15.7%) exhibited the biofilm-producing phenotype. In contrast, only 5 (4.6%) of the 108 *stx*-positive isolates were classified as biofilm producers, with the majority (103 isolates) being not able to form biofilm. Although the *stx* gene itself has no direct role in biofilm production, the insertion of the toxin-encoding prophage has been linked in the literature to the reduction or inhibition of biofilm formation, an effect that depends on the specific integration site within the bacterial chromosome [16]. The most well-documented mechanism involves the integration of the *stx* prophage into the *mlrA* gene, a transcriptional regulator that acts as an activator of *csgD* expression [13,16]. The *csgD* gene acts as a master regulator of curli and cellulose synthesis. Therefore, the disruption of *mlrA* by the prophage results in the silencing of the *CsgD*-dependent biofilm cascade, even when the *csg* and *bcs* structural genes are fully intact [14].

Results by toxin subtype and *mlrA* locus status demonstrated that isolates concurrently carrying *stx1* and *stx2* exhibited the lowest biofilm-positivity rate (3.3%), whereas non-*stx* isolates reached 16.5% biofilm-positivity (Table 1). This difference is consistent with a phage-mediated suppression model, wherein strains harboring multiple lysogenic prophages may have a higher probability of accumulating insertion events within *mlrA* or within prophage-encoded regulators that indirectly downregulate *csgD*, thereby increasing the likelihood of functionally silencing the *CsgD*-dependent biofilm cascade [13,14]. Furthermore, even among isolates classified as ‘free’ (lacking direct or proximal prophage insertion at the *mlrA* locus), several *stx*-positive O157:H7 strains retained a low biofilm-forming capacity compared to *stx*-negative strains. This observation is consistent with the notion that lysogeny can modulate the *CsgD*-dependent biofilm phenotype through regulatory mechanisms independent of intragenic *mlrA* disruption [45].

The phage-proximal group (prophage near the *mlrA* gene but not inserted into it) displayed a heterogeneous pattern: *stx1*-only isolates exhibited a higher biofilm-positivity rate than the equivalent free group, while phage-proximal *stx2*-only and *stx1*&*stx2* isolates recorded no biofilm production (Table 1). For the non-*stx* phage-proximal group, a reduction in biofilm formation was observed compared to the non-*stx* free group (11.1% vs. 16.5%), suggesting that the mere proximity of a phage element to the *mlrA* locus can exert a regulatory effect through adjacency, even without direct disruption of the gene. However, the overall biofilm-positivity rate remained similar between the free and phage-proximal groups (10.4% vs. 9.5%), indicating that *mlrA* locus status is not the sole determinant of the evaluated phenotype.

The analysis of the association between *rpoS* mutational status and biofilm formation (Table 2) revealed no statistical significance (*p* = 0.354). This finding contrasts with expectations based on the published literature. This discrepancy may be partially explained by the heterogeneous composition of the strain collection. Previous studies have demonstrated that *rpoS* mutations limit curli expression and biofilm formation, particularly in O157:H7 strains, where such mutations are highly prevalent [45]. In a dataset characterized by a wide diversity of serogroups and STs, alternative biofilm regulatory mechanisms may predominate, thereby diluting the overall influence of the *rpoS* mutation.

Accordingly, the association between *rpoS* status and the O157:H7 serogroup was evaluated. The results revealed a scenario completely opposite to that observed across all isolates (Table 3). All 71 O157:H7 isolates harbored at least one mutation in the *rpoS* gene, whereas none of the 77 isolates with an intact *rpoS* gene belonged to this serogroup (Table 3). This correlation is not coincidental; indeed, *rpoS* mutations in *E. coli* are widely documented as genomic signatures [46,47], resulting from specific selective pressures [48]. Therefore, in O157:H7, biofilm suppression may be mediated by redundant and concurrent mechanisms: the disruption of *mlrA* by the *stx* prophage and mutations within *rpoS*, both converging on the repression of *csgD*. This redundancy suggests that biofilm limitation in this lineage may be multifactorial, possibly as an adaptive consequence of coevolution with highly integrative *stx* phages.

In addition to the analysis of *rpoS* mutations, a comparison of mean SNP counts per gene involved in curli (*csg*) and cellulose (*bcs*) synthesis was conducted. When comparing *stx*-positive and *stx*-negative isolates, the results demonstrated that virtually all genes within the *csg* (curli) and *bcs* (cellulose) operons accumulated significantly more SNPs in *stx*-positive strains. The exceptions were the *bcsG* gene, which showed no significant difference, and *csgF*, which exhibited a trend opposite to the others (Table 4). A careful interpretation suggests that in STEC strains, where the *csgD* pathway is chronically suppressed by prophage insertion into *mlrA* and mutations within *rpoS*, these structural curli and cellulose genes are less expressed and can therefore accumulate mutations without an immediate fitness cost.

The lack of a significant difference for *bcsG* is biologically relevant. This gene encodes a phosphoethanolamine transferase responsible for the post-synthetic modification of cellulose, a function with broader physiological roles that extend beyond biofilm production alone [49]. Its conservation across *stx*-positive and *stx*-negative isolates suggests that selective pressure is maintained regardless of toxigenic status. Conversely, the contrasting trend observed for *csgF* (which harbored fewer SNPs in *stx*-positive strains) may reflect unique characteristics of this accessory gene in curli fiber assembly, although the precise mechanism remains to be fully elucidated.

In the present study, no association was observed between the overall SNP load and the biofilm phenotype (biofilm-positive vs. biofilm-negative). This dissociation supports the interpretation that biofilm limitation in STEC may be predominantly regulatory rather than structural in nature. For instance, the *csgA* and *bcsA* genes, along with other extracellular matrix components, may be present and partially intact at the sequence level, yet remain transcriptionally silenced by the suppression of *csgD*.

### 3.2. Determination of Biofilm Genes

**Figure 2:**
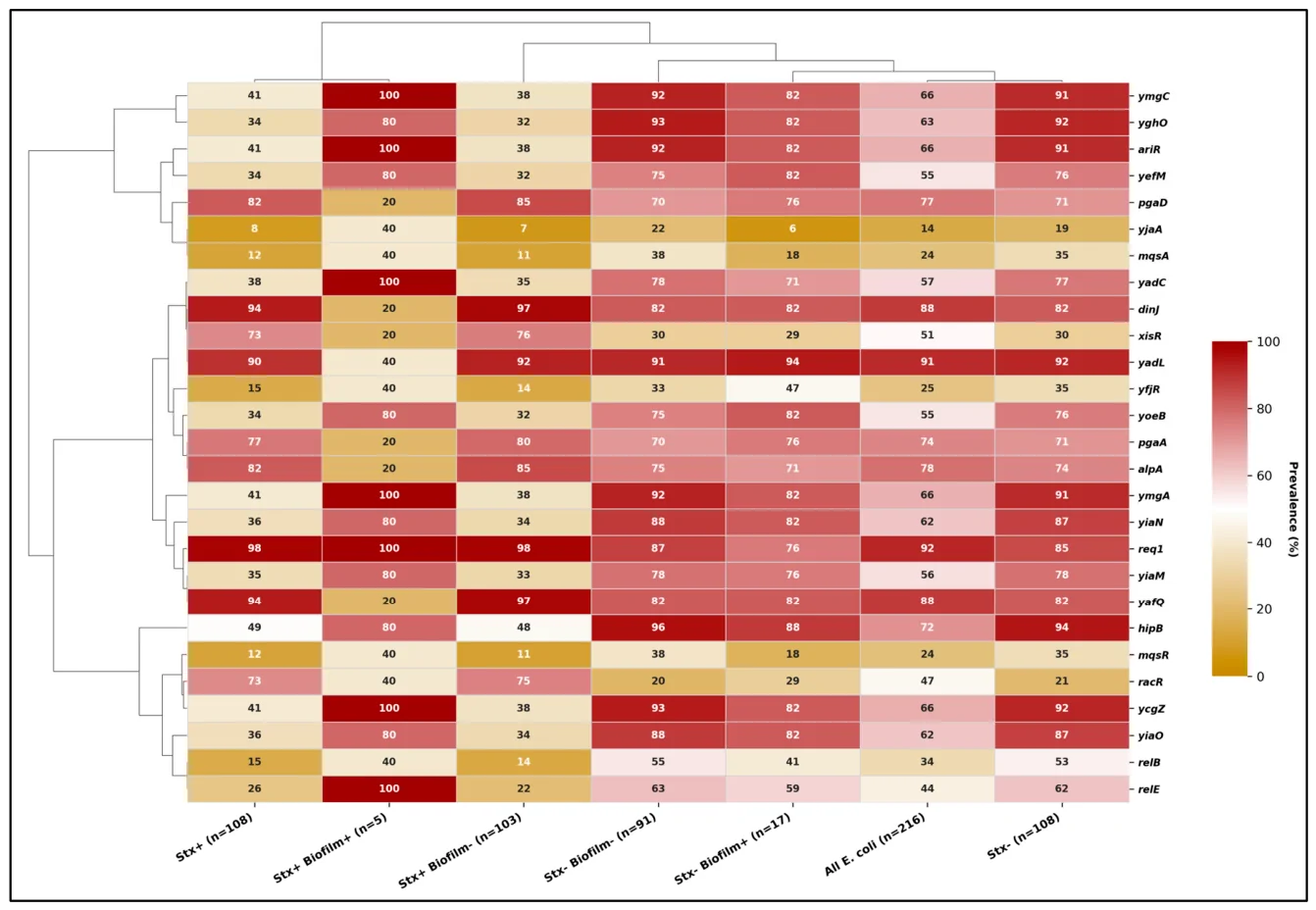
Prevalence profile and hierarchical clustering of genes associated with biofilm formation, stress response, and toxin-antitoxin systems across *Escherichia coli* subpopulations. **Legend:** Heatmap with hierarchical clustering dendrograms based on gene presence/absence. Rows represent the 27 evaluated regulatory and structural genes, whereas columns display the subpopulations of isolates stratified by *stx* status and phenotypic biofilm-forming capacity. The numerical values inside the cells and the color gradient indicate the percentage prevalence (%) of the gene within each group (scale from yellow [0%] to dark red [100%]).

The heatmap (Figure 2) illustrates the prevalence (%) of biofilm-associated genes across seven groups (1. *stx*+; 2. *stx*+ biofilm−; 3. *stx*+ and biofilm−; 4. *stx*− and biofilm−; 5. *stx*− and biofilm+; 6. all *E. coli*; and 7. *stx*−). The overall pattern highlighted that the *stx*+ and biofilm+ group exhibited the sharpest contrast, displaying the darkest values (red) for several genes, as well as the lightest values (yellow) for others, thereby revealing a distinct genomic profile for this subgroup.

Among the genes retained after prevalence-based filtering and visualized in the heatmap, with hierarchical clustering, a subset comprising *ymgC*, *yghO*, *ariR* (=*ymgB*), *yefM*, *yadC*, *yoeB*, *ymgA*, *yiaN*, *req1*, *hipB*, *ycgZ*, *yiaO*, and *relA* showed higher prevalence in *stx*-positive, biofilm-positive isolates compared to *stx*-positive, biofilm-negative isolates (Figure 2). This pattern suggests that biofilm formation in STEC may be mediated by pathways distinct from classical chromosomal regulatory networks, involving chemical signaling regulation, toxin-antitoxin (TA) systems, and minor fimbrial adhesins.

The genes *ymgA*, *ymgB* (renamed *ariR*: acid-resistance influenced by indole by [50]), and *ymgC* constitute a regulatory locus whose deletion in *E. coli* K-12 results in a 4- to 9-fold increase in biofilm formation, respectively [51]. Furthermore, *ycgZ* is co-encoded within the same regulatory locus and, together with *YmgA* and *AriR*, forms a complex that interacts with *RcsC* to modulate biofilm formation and acid resistance. Notably, mutants within this operon, including *ycgZ*, exhibit altered biofilm phenotypes [52]. Additional structural studies have demonstrated that AriR/YmgB is a dimer exhibiting structural homology to the *Hha* protein [50]. This gene functions as a global regulator that modulates the expression of multiple genes involved in biofilm formation, motility, and acid resistance, partly through the integration of indole signaling and the stress response [53]. Furthermore, elevated indole levels repress biofilm formation in *E. coli*, partially via *SdiA* [54]. The high prevalence of these genes involved in biofilm repression may reflect that the basal expression of these regulators is upregulated as a homeostatic response to the stress imposed by *stx* prophages. Furthermore, the *ycfR* gene encodes an outer membrane protein involved in the stress response, thereby modulating indole synthesis [55]. Its deletion has previously been linked to a reduction in biofilm formation in *E. coli*, alongside the downregulation of acid resistance genes [55].

The HipA/HipB system is a well-characterized type II toxin-antitoxin (TA) system in *E. coli* [56]. The HipA toxin functions as a serine/threonine kinase that inactivates the aminoacyl-tRNA synthetase GltX, thereby arresting translation and triggering the stringent response [57]. This arrest induces cellular dormancy, which can trigger the central physiological state crucial for the formation of persister cells [58]. The HipB antitoxin neutralizes HipA under favorable environmental conditions, but the presence of this system indicates the capacity to alternate between planktonic growth and the persister state [59]. 

The *yefM* and *yoeB* genes, which constitute another type II TA system, were also present. Studies in other species have demonstrated that this system independently promotes colonization and is associated with biofilm formation in in vivo infection models [60]. The *YoeB* toxin is an endoribonuclease that, upon activation, cleaves ribosome-bound mRNAs, thereby inducing bacteriostasis and facilitating entry into a quiescent state compatible with biofilm persistence [61].

The *relA* gene encodes (p)ppGpp synthetase I, which is responsible for producing the alarmone (p)ppGpp during the stringent response under stress conditions. In *E. coli*, elevated (p)ppGpp levels globally reprogram transcription and metabolism, promote the activation of stress regulators such as *rpoS*, and are associated with enhanced biofilm formation and antibiotic tolerance in both planktonic populations and biofilms [62]. Under conditions of nutritional, oxidative, or genetic element integration-related stress (such as prophages), the *relA* gene converts GDP/GTP into ppGpp, which (i) reduces the growth rate, and (ii) activates *rpoS* (σ^S^, a key regulator of biofilm formation and the stress response) [62]. Therefore, we hypothesize that chronic stress environments, such as those imposed by the maintenance of chromosomally integrated *stx* prophages, may favor the recurrent activation of the stringent response and elevated, *relA*-mediated (p)ppGpp levels. This establishes a physiological state characterized by slow growth and an active stress response, which is well-known to promote biofilm formation and antibiotic tolerance. The link between the stringent response and biofilm development has been directly demonstrated in *E. coli relA* and *spoT* mutants, which exhibit significantly reduced biofilm formation [63].

Finally, *YadC* is the adhesive tip subunit of Yad fimbriae in *E. coli*, a chaperone-usher system involved in initial surface adhesion and in phenotypes associated with biofilm formation and persistence [6,64,65]. The higher prevalence in *stx*-positive, biofilm-positive isolates suggests that the adhesive contribution of the Yad pathway may serve as a differential determinant of biofilm-forming capacity within this subgroup, complementing regulation mediated by indole/AI-2 and TA systems.

### 3.3. Antimicrobial Resistance Genes, Plasmid Presence and Biofilm Production

**Figure 3:**
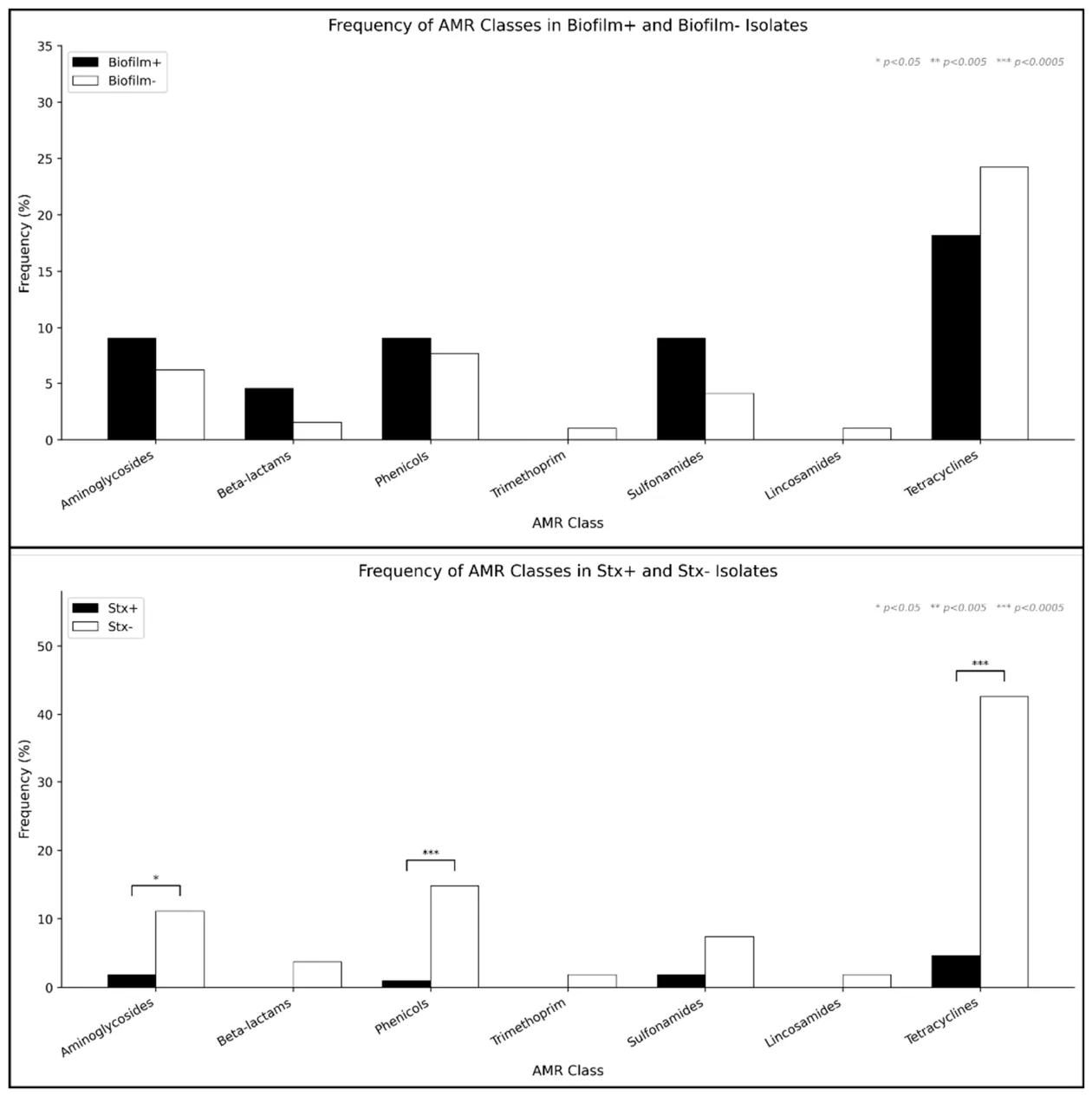
Comparative frequency of antimicrobial resistance (AMR) classes in *Escherichia coli* isolates categorized by biofilm phenotype and virulence profile (*stx*). **Legend:** Percentage distribution of AMR determinants in bacterial subpopulations. The upper panel delineates the frequency of resistance classes between biofilm-producing (Biofilm+, black bars) and non-producing isolates (Biofilm−, white bars), highlighting the absence of statistically significant disparities between the two phenotypic profiles. The lower panel compares AMR frequency between Shiga toxin-producing isolates (*stx*+, black bars) and non-carriers (*stx*−, white bars). Brackets and asterisks indicate statistically significant differences determined by Fisher’s exact tests (*p* < 0.05; *p* < 0.0005).

The results revealed a consistent pattern characterized by a lack of statistically significant differences between biofilm-positive and biofilm-negative isolates across all AMR classes (Figure 3). Comparable frequencies were observed for aminoglycosides (~9% vs. ~6%), phenicols (~9% vs. ~7%), sulfonamides (~9% vs. ~4%), and tetracyclines (~18% vs. ~24%), whereas the prevalence remained remarkably low for beta-lactams, trimethoprim, and lincosamides in both groups. Consequently, antimicrobial resistance was not associated with the biofilm phenotype in the present study. Biofilm formation and antimicrobial resistance are frequently observed in tandem, as growth within a biofilm architecture enhances physiological tolerance to antimicrobials [66]. The lack of a stronger association between biofilm formation and specific antimicrobial resistance profiles reinforces the hypothesis that biofilms are composed of heterogeneous cellular subpopulations. This architecture facilitates functional sharing and a clear division of labor among the resident cells, whereby certain subpopulations specialize in biofilm matrix development while others exhibit enhanced antimicrobial resistance [67].

Furthermore, the high prevalence of tetracycline resistance (*tetA*, *tetB*, and *tetM*) is likely in *E. coli* isolates from animal and environmental sources, as these determinants are frequently carried on broadly disseminated conjugative plasmids [68,69]. Overall, the *stx*-positive isolates in the present study exhibited a lower frequency of antimicrobial resistance across all classes. This lower frequency of resistance may be consistent with the evolutionary trajectories of STEC lineages, which frequently demonstrate a trade-off resulting in a low long-term retention of resistance plasmids [70]. Given that the STEC genome is characterized by a high degree of polylysogeny, the subsequent acquisition of large conjugative plasmids may impose an additional metabolic burden (fitness-cost), potentially triggering genomic instability in the absence of direct antimicrobial selective pressure. A clear disparity and evolutionary trade-off in *E. coli* have previously been highlighted in the literature, demonstrating that lineages specialized in the accumulation of virulence prophages face adaptive constraints regarding the stable maintenance of multidrug-resistant mobile genetic elements [71].

Following the analysis of resistance by antimicrobial class, we evaluated the distribution and frequency of plasmid replicons in relation to the biofilm phenotype. Notably, the IncFIB (AP001918) replicon was more prevalent in biofilm-negative isolates than in biofilm-positive ones (Figure 3). This incompatibility family is typically associated with large, conjugative plasmids harboring antimicrobial resistance genes, including beta-lactamases, as well as robust plasmid maintenance systems [72]. Therefore, the higher prevalence of these plasmids within the biofilm-negative phenotype may be related to the fitness cost they impose on the host. In isolates that already harbor integrated *stx* prophages (the metabolic cost of which is inherently high) the additional presence of a large IncFIB plasmid may compromise the energetic investment required for biofilm formation.

**Figure 4:**
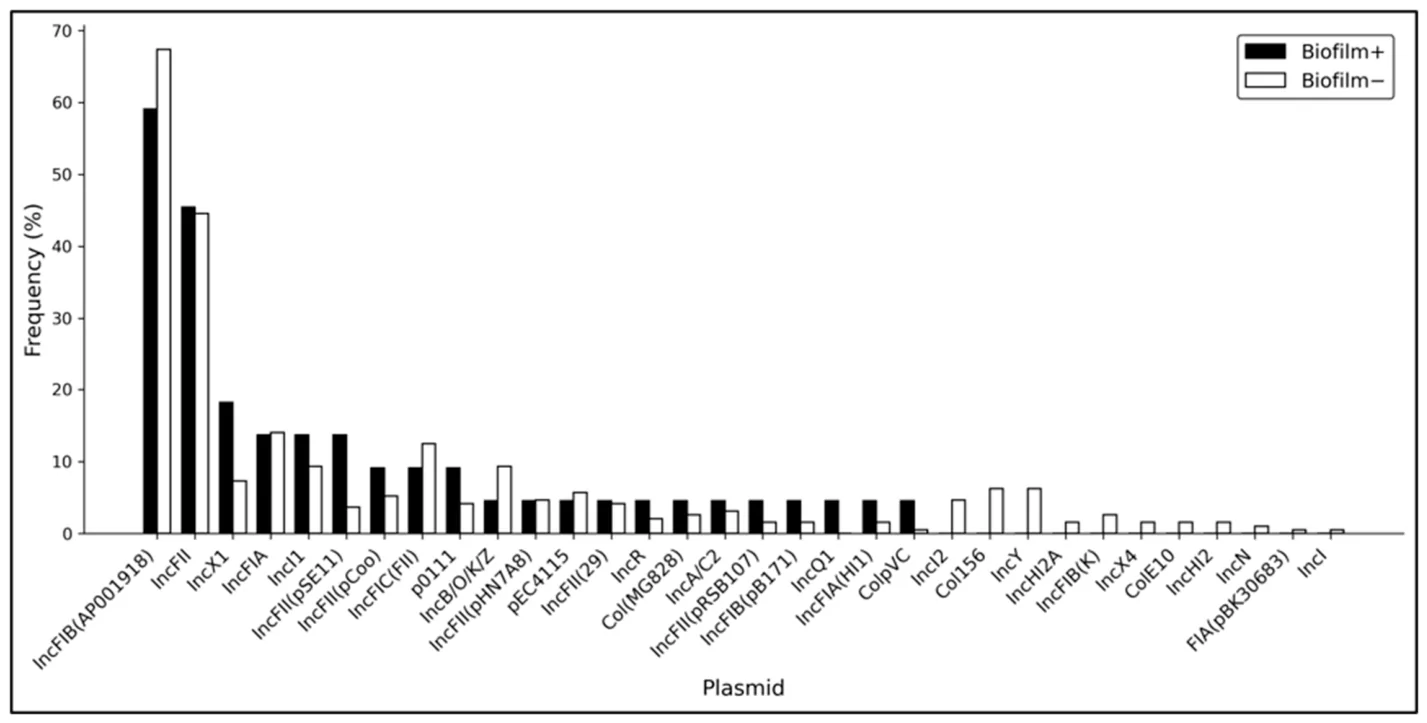
Relative frequency of plasmid replicons in *Escherichia coli* isolates categorized by biofilm-forming capacity. **Legend:** Epidemiological distribution of plasmid incompatibility (Inc) profiles. Black bars represent the percentage frequency (%) of replicons in biofilm-producing isolates (Biofilm+), whereas white bars indicate the frequency in the non-producing group (Biofilm−). The X-axis delineates the 32 replicons and specific variants identified by PlasmidFinder.

Conversely, the IncX1 replicon was more prevalent in biofilm-positive isolates (Figure 4). This result agrees with the literature, where the IncX1 plasmid pOLA52 (the prototype of this incompatibility group) was shown to encode type III fimbriae via the *mrk* operon, which acts as a direct promoter of both biofilm formation and conjugative transfer frequency in *Enterobacteriaceae* [18]. Furthermore, the IncX1 backbone contains an active partition system and a toxin-antitoxin maintenance system, which ensure plasmid stability within the cell and may indirectly contribute to the persistence of the biofilm phenotype in carrier isolates [73].

Finally, the IncFII(pSE11) plasmid replicon also exhibited a differential distribution between biofilm-forming and non-forming isolates. The IncFII(pSE11) replicon is named after the pSE11 plasmid, originally described in the commensal *E. coli* strain SE11 (O152:H28, phylogroup B1), which was isolated from the feces of a healthy adult [74]. Unlike classical IncFII plasmids that harbor antimicrobial resistance genes, the SE11 genome and its plasmids exhibit no similarity to either pathogenic virulence genes or resistance determinants. However, they are remarkably enriched in genes encoding fimbriae and autotransporters, surface-associated structures that contribute directly to intestinal cell adhesion and stable colonization. Comparative analyses of large IncF plasmids confirm that this category of replicons frequently carries colonization factors, adhesion systems, and autotransporters in both commensal and pathogenic *E. coli* [72]. Therefore, the higher prevalence of this replicon in biofilm-positive isolates is biologically plausible, as *E. coli* fimbriae and autotransporters often exert overlapping functions in sustained mucosal colonization and the adhesion required for biofilm formation on both biotic and abiotic surfaces [75,76,77].

### 3.4. Multiple Correspondence Analysis of Non-stx Gene Profiles

The complete dataset used for the analysis included 216 isolates and 670 binary gene variables, in addition to metadata variables and supplementary variables used for interpretation. The distribution of isolates according to *stx* presence was balanced, with 108 isolates classified as *Stx*_0 and 108 as *Stx*_1. Regarding biofilm production, 194 isolates were classified as non-producers and 22 as producers.

**Figure 5:**
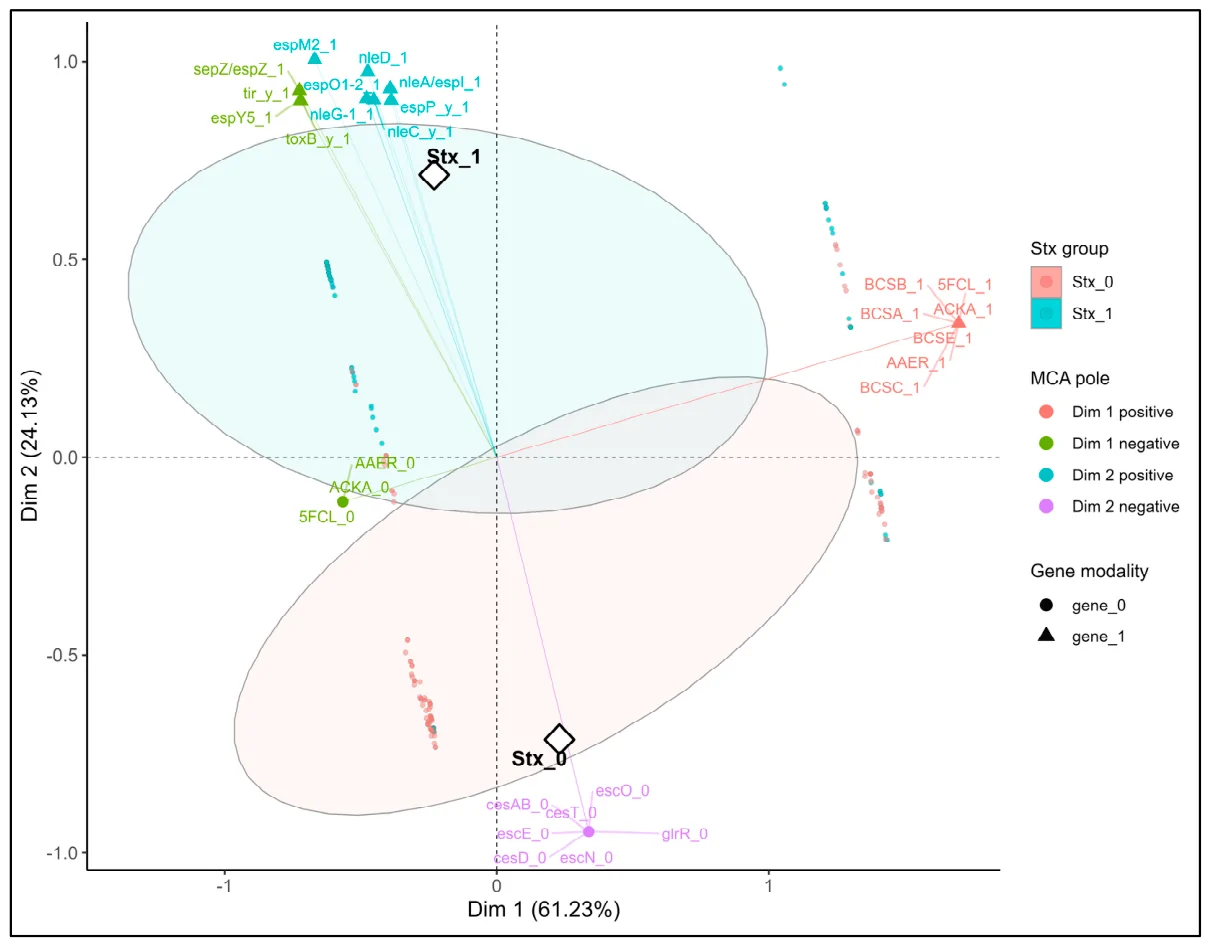
MCA biplot of non-*stx* gene profiles with supplementary projection of *stx* presence. **Legend:** The multiple correspondence analysis (MCA) was constructed using 304 selected non-*stx* genes as active variables. Isolates are represented according to their coordinates on the first two dimensions and grouped according to the supplementary variable *stx*_presence (*Stx*_0 or *Stx*_1). Gene modalities are presented as absence (gene_0) or presence (gene_1). Dimension 1 primarily represented a global contrast between presence and absence modalities of genes such as *ackA*, *aaeR*, *bcsA*, *bcsB*, *bcsC*, and *5fcl*. Dimension 2 showed a stronger visual relationship with *stx*_presence, with presence modalities of genes such as *espM2*, *nleD*, *nleA*/*espI*, *espP_y*, and *espO1-1* located near the *Stx*_1 region, and absence modalities of genes such as *cesT*, *cesAB*, *escE*, *cesD*, *escN*, and *glrR* located near the *Stx*_0 region. The displayed gene modalities correspond to the seven modalities with the highest score at each factorial pole, objectively selected based on the coordinates and contributions of the modalities on the first two dimensions. The proximity between supplementary categories and gene modalities should be interpreted as an exploratory pattern of co-occurrence on the factorial plane, rather than as a causal relationship. Complete MCA results are provided in Supplementary Table S4, and the modalities selected for the final biplot are described in Supplementary Table S5.

Following the calculation of the presence frequency for each gene, 308 genes were present in between 25% and 75% of the isolates and were initially retained due to their variability for multivariate analysis. Among these, genes starting with *stx* were excluded from the active matrix, as the *stx*_presence variable was utilized as a supplementary grouping category. Thus, the final active MCA matrix was composed of 304 non-*stx* genes, encoded as absence (gene_0) and presence (gene_1) modalities. Therefore, the final analytical matrix integrated the selected non-*stx* genes as active variables, while *stx*_presence, biofilm production, and serogroup were included as supplementary variables (Figure 5).

As a complementary step to the multivariate analysis, exploratory associations between *stx* presence and the variables biofilm production and serogroup were evaluated, considering the low total number of biofilm-producing isolates, the high number of serogroup categories, and the unequal distribution of isolates among these categories as limitations of the analysis. Biofilm production showed an exploratory association with *stx* presence, with a lower frequency of producing isolates among *Stx*_1 compared to *Stx*_0. 

Tests performed at the gene level indicated that 130 non-*stx* genes remained associated with *stx* presence. Among the associated genes that also contributed to the interpretation of the biplot (Figure 5), higher frequencies were observed in *Stx*_1 isolates for *espO1-1* (88/108 in *Stx*_1 and 5/108 in *Stx*_0; adjusted *p* = 1.19 × 10^−27^; Cramér’s V = 0.776), *espP_y* (90/108 in *Stx*_1 and 9/108 in *Stx*_0; adjusted *p* = 1.74 × 10^−26^; Cramér’s V = 0.753), *espM2* (73/108 in *Stx*_1 and 0/108 in *Stx*_0; adjusted *p* = 3.26 × 10^−24^; Cramér’s V = 0.714), *nleD* (78/108 in *Stx*_1 and 5/108 in *Stx*_0; adjusted *p* = 3.57 × 10^−23^; Cramér’s V = 0.695), and *nleA*/*espI* (83/108 in *Stx*_1 and 10/108 in *Stx*_0; adjusted *p* = 1.26 × 10^−22^; Cramér’s V = 0.683).

Other genes related to the modalities highlighted in the biplot (Figure 5) also exhibited higher frequency in *Stx*_1 isolates, including the genes *cesT*, *cesAB*, *escE*, *cesD*, *escN*, and *glrR*, which were detected in 94/108 *Stx*_1 isolates and 21/108 *Stx*_0 isolates (*p* = 1.26 × 10^−22^; Cramér’s V = 0.677). These results assist in interpreting the opposition observed in the factorial plane, as the presence of these variables was more frequent in isolates harboring the *stx* gene (*Stx*_1), whereas their absence modalities contributed to characterizing the region near *Stx*_0 in the MCA. Thus, the gene-level association tests reinforce that the differences between *Stx*_0 and *Stx*_1 isolates involve a broader set of non-*stx* genes beyond the mere presence or absence of *stx* genes.

The combination of bivariate tests and multiple correspondence analysis supports the interpretation that *Stx*_0 and *Stx*_1 isolates differ in broader patterns of gene composition, rather than merely by the presence or absence of *stx* genes. Nevertheless, these results should be interpreted considering the exploratory nature of the analyses, the potential influence of population structure represented by serogroups, and the partial overlap observed among individuals in the biplot. Therefore, the findings indicate patterns of co-occurrence and multivariate structure, but do not allow for inferences of causality, direct functionality, or independence from the genomic background of the isolates.

Analyzing the results beyond the presence or absence of *stx* genes, our multivariate and bivariate analyses reveal that *stx*-positive (*Stx*_1) and *stx*-negative (*Stx*_0) isolates were defined by distinct genomic landscapes. Our result identified a set of non-*stx* genes significantly associated with the presence of *stx*; specifically, effectors such as *espM2*, *nleD*, *nleA*/*espI*, *espP_y*, and *espO1-1* exhibited a strong positive correlation with *stx*-positive isolates. These same genes were also projected as presence modalities near the Stx_1 supplementary category in the MCA, indicating agreement between the gene-level association tests and the factorial plane. This result corroborated the large effect size and high statistical significance. These patterns of co-occurrence, visualized through MCA, indicate that the genomic architecture of these populations is structured in such a way that *stx* acquisition is linked to a conserved set of accessory genes, or at least co-occurs with a broader repertoire of non-*stx* genes in the present dataset, a phenomenon consistent with the organized evolution of the *E. coli* pangenome via horizontal gene transfer. The association of these *stx*-positive lineages with a specific repertoire of non-LEE effectors suggests that these strains share an evolutionary trajectory where the acquisition of mobile virulence elements is concomitant with the stabilization of essential pathogenicity factors [78]. In contrast, the Stx_0 region of the factorial plane was mainly characterized by absence modalities of genes such as *cesT*, *cesAB*, *escE*, *cesD*, *escN*, and *glrR*. This does not indicate that these genes were more frequent in Stx_0 isolates; rather, it indicates that the absence modalities of these genes contributed to the region opposite to Stx_1 in the MCA. In this context, the projection of *stx*-negative isolates near the absent modalities, characterized by the frequent presence of type III secretion system (T3SS) components, reflects a divergence in genome structuring, where bacterial plasticity allows the genetic backbone to vary independently of the toxin status [79]. Although these findings do not imply direct causality, they demonstrate that *stx* acts as a diagnostic marker for deeper genomic disparities, within the context of this isolate collection and as a supplementary grouping variable in the present analysis, reflecting evolutionary lineages with different adaptive strategies. Therefore, these findings support the interpretation that *stx* presence is associated with broader differences in accessory gene composition and multivariate structuring among the isolates, within the genomic context evaluated in this study.

Furthermore, the results demonstrate that Dimension 1 (Figure 5) explained 61.23% of the total observed variation, serving as the main axis of differentiation among the isolates’ genetic profiles. It highlighted a contrast in the presence of a specific group of genes, such as *ackA*, *aaeR*, *bcsA*, *bcsB*, *bcsC*, and *5FCL*. This gene separation revealed a fundamental genetic structure within our sample and demonstrated partitioning into distinct groups. It is important to highlight that this variation occurred independently of the presence of *stx* genes. Thus, this axis reflected an essential difference in the genomic background of all sequences. Therefore, the results suggest a fundamental divergence in terms of metabolism and colonization capacity among the isolates. The clustering of the *bcsA*, *bcsB*, and *bcsC* genes is significant because they comprise the bcs operon, which is essential for cellulose biosynthesis, a primary component of the bacterial extracellular matrix and fundamental for biofilm formation and environmental resistance [80]. Simultaneously, the *ackA* gene (acetate kinase) plays a central role in energy metabolism and carbon flux regulation, which is essential for the adaptation of *E. coli* to different nutrient sources and growth conditions [81]. Thus, the result shown in Figure 5 illustrates a differentiation between lineages with distinct survival strategies: one group with a more robust potential for cellulose synthesis and metabolic adaptation, and another that lacks this structural genetic apparatus.

## 4. Limitations of the Study

Although this study leveraged a large genomic dataset to investigate differences in biofilm production between STEC and non-STEC isolates, several limitations should be considered when interpreting the findings. First, biofilm formation was evaluated exclusively on polystyrene under the experimental conditions adopted in this study. Although polystyrene is widely used for in vitro biofilm screening, biofilm development is influenced by surface composition, temperature, nutrient availability, incubation time, and other environmental factors. Consequently, the inability of an isolate to form a biofilm in this model does not exclude its potential to colonize other surfaces relevant to animal production and food-processing environments, such as stainless steel, glass, rubber, concrete, or wood. Conversely, biofilm formation on polystyrene may not directly predict persistence under industrial conditions.

Second, the observed frequency of single nucleotide polymorphisms in biofilm-associated loci, including genes within the *csg* and *bcs* clusters, should not be interpreted as direct evidence of impaired gene function. The functional consequences of these variants depend on their genomic position, their predicted effect on protein structure, and their potential influence on regulatory regions. Moreover, biofilm formation is a complex and multifactorial phenotype that may be affected by gene regulation, epistatic interactions, accessory genes, and environmental signals. Therefore, complementary analyses, including variant-effect prediction, gene-expression assays, transcriptomic approaches, targeted mutagenesis, and functional complementation, would be necessary to establish causal relationships between specific genomic variants and biofilm phenotypes.

Third, the whole-genome sequencing approach used in this study primarily enabled the identification of known core and accessory genes potentially associated with biofilm formation. Gene detection and variant identification may, however, be influenced by sequencing coverage, genome assembly quality, annotation accuracy, reference-database completeness, and the thresholds used to define gene presence or absence. In addition, the analysis did not comprehensively assess regulatory sequences, structural variants, gene expression, or other molecular mechanisms that may contribute to the observed phenotypic variation.

Finally, because the collection comprised isolates obtained from cattle feedlot environments, the generalizability of the findings to isolates from other production systems, geographic regions, animal hosts, food matrices, or processing environments may be limited. Thus, the present results should be interpreted as an exploratory genomic assessment of cattle feedlot-derived isolates rather than as definitive evidence of the molecular mechanisms governing biofilm formation. Nevertheless, the study provides a relevant genomic framework for prioritizing candidate genes and variants for subsequent functional validation under a broader range of experimentally and industrially relevant conditions.

## 5. Conclusions

The present study identified a potential evolutionary and regulatory trade-off in bovine *Escherichia coli* isolates, wherein the acquisition and maintenance of *stx* prophages were consistently associated with a marked suppression of biofilm-forming capacity on polystyrene. This inhibition of the phenotype in STEC lineages is likely regulatory rather than structural in nature, occurring through redundant and multifactorial mechanisms. Notably, intragenic or flanking disruptions of the *mlrA* locus, coupled with the universal presence of mutations in the *rpoS* gene in the O157:H7 serogroup, converge to silence the *csgD*-dependent cascade. Consistent with this transcriptional blockade, the structural genes of the extracellular matrix (*csg* and *bcs*) accumulate significantly more mutations (SNPs) in *stx*-positive lineages, likely due to the relaxation of direct selective pressure on these regions.

Furthermore, the analysis of these variable accessory genes suggests that biofilm-forming capacity in *E. coli* emerges from decentralized and independent genomic trajectories, driven by the high genomic plasticity of the species, which complicates the identification of a universal genetic marker for biofilm production. This functional independence is further reflected in the absence of a direct association between antimicrobial resistance (AMR) classes and the biofilm phenotype. While lysogeny and large resistance-carrying plasmids (such as IncFIB) may impose fitness constraints affecting extracellular polymeric substance synthesis in STEC, specific plasmid replicons involved in colonization and cellular fitness, such as IncX1 and IncFII(pSE11), may facilitate the biofilm-positive phenotype.

Finally, the findings of the present study highlight the epidemiological risk posed by non-STEC lineages, which retained more intact biofilm regulatory axes and exhibited a higher capacity for environmental persistence. Given that STEC and non-STEC strains can share the same clinically relevant classic serogroups (e.g., O157, O26, O103, O121, and O145), this constitutes a persistent and potentially underestimated reservoir within the beef production chain. Thus, epidemiological surveillance and sanitary control programs should expand their diagnostic tools beyond the simple molecular detection of the *stx* gene, integrating the assessment of regulatory axes and cellular persistence to effectively mitigate the risks of cross-contamination in food products.

## Figures and Tables

**Table 1:** *mlrA* site and phage inclusion or proximity as compared to biofilm-forming capacity. ‘Free’ isolates lack prophage insertion sites within or proximal to *mlrA*.

Variable	Free (n = 192)	Free Biofilm+	Phage-Proximal (n = 21)	Phage-Proximal Biofilm+
*stx1* only	21 (10.9%)	1/21 (4.8%)	6 (28.6%)	1/6 (16.7%)
*stx2* only	14 (7.3%)	1/14 (7.1%)	2 (9.5%)	0/2 (0.0%)
*stx1* & *stx2*	60 (31.2%)	2/60 (3.3%)	4 (19.0%)	0/4 (0.0%)
Non-*stx*	97 (50.5%)	16/97 (16.5%)	9 (42.9%)	1/9 (11.1%)
**Total Biofilm+**	**20 (10.4%)**		**2 (9.5%)**	

**Table 2:** Association between *rpoS* mutational status and biofilm production in *E. coli* isolates (n = 214).

*rpoS* Status	Biofilm Positive	Biofilm Negative	Total
*rpoS* intact	10	67	77
*rpoS* mutated	12	125	137
**Total**	22	192	214

**Legend:** Fisher’s exact test: OR = 1.555 (95% CI: 0.638–3.787); *p* = 0.354 (ns).

**Table 3:** Association between *rpoS* mutational status and O157:H7 serogroup in *E. coli* isolates (n = 214).

*rpoS* Status	Non-O157:H7	O157:H7	Total
*rpoS* intact	77	0	77
*rpoS* mutated	66	71	137
**Total**	143	71	214

**Table 4:** Comparison of mean SNP counts per gene between *stx*-positive and *stx*-negative *E. coli* isolates.

Gene	Mean SNPs (*stx*−)	Mean SNPs (*stx*+)
*csgA*	6.06	16.29 *
*csgB*	0.90	1.59 *
*csgC*	3.03	4.53 *
*csgD*	3.67	5.22 *
*csgE*	1.06	2.15 *
*csgF*	1.65	0.69 *
*csgG*	7.94	10.93 *
*bcsA*	34.38	40.71 *
*bcsB*	13.67	19.67 *
*bcsC*	32.71	38.08 *
*bcsE*	5.10	5.94 *
*bcsF*	0.26	0.63 *
*bcsG*	10.69	10.05 ^ns^
*bcsQ*	8.18	10.63 *
*bcsZ*	5.64	9.14 *

**Legend:** * *p* < 0.05 (Mann–Whitney U test); ^ns^ not significant. Gene names are italicized following standard nomenclature.

## Data Availability

The data presented in this study are openly available in NCBI at https://www.ncbi.nlm.nih.gov/bioproject (accessed on 22 July 2026), PRJNAS1166229, PRJNA1038721, PRJNA601484, PRJNA870153, and PRJNA846735.

## Supplementary Materials

The following supporting information can be downloaded at https://www.mdpi.com/article/10.3390/pathogens15080807/s1, Table S1: Dataset Diagnostic and Multiple Correspondence Analysis (MCA) Matrices Parameters (including complete dataset overview, intermediate frequency gene filtration, and final parameters defining the active MCA matrix); Table S2: Genomic Frequency Distribution of Virulence, Antimicrobial Resistance, and Biofilm-Associated Genes Across Bovine *Escherichia coli* Isolates (STEC and non-STEC) and Serogroups; Table S3: Bivariate Statistical Association Tests (Pearson Chi-Square and Simulated Fisher’s Exact Tests) Cross-Tabulating *stx*-Presence, Biofilm Production Capacity, Serogroups, and Genomic Markers with False Discovery Rate (FDR) Control; Table S4: Contribution and Cosine Square Coordinates for Modalities and Top Target Modalities Defining the Main Geometric Poles in the Final MCA Biplot Dimensions; Table S5: Selected Modalities for Final MCA Biplot; Dataset S1: Complete Aggregated Source Genomic and Phenotypic Input Matrix for the 216 Bovine *Escherichia coli* Isolates; Dataset S2: Final Analytical Transformed Modality Binary Dataset Utilized Directly for Multiple Correspondence Analysis (MCA) Computation.
